# Lixisenatide Reduces Chylomicron Triacylglycerol by Increased Clearance

**DOI:** 10.1210/jc.2018-01176

**Published:** 2018-09-11

**Authors:** Martin B Whyte, Fariba Shojaee-Moradie, Sharaf E Sharaf, Nicola C Jackson, Barbara Fielding, Roman Hovorka, Jeewaka Mendis, David Russell-Jones, A Margot Umpleby

**Affiliations:** 1 Faculty of Health and Medical Sciences, University of Surrey, Guildford, United Kingdom; 2 Diabetes Modelling Group, Institute of Metabolic Science, University of Cambridge, Cambridge, United Kingdom

## Abstract

**Context:**

Glucagon-like peptide-1 (GLP-1) agonists control postprandial glucose and lipid excursion in type 2 diabetes; however, the mechanisms are unclear.

**Objective:**

To determine the mechanisms of postprandial lipid and glucose control with lixisenatide (GLP-1 analog) in type 2 diabetes.

**Design:**

Randomized, double-blind, cross-over study.

**Setting:**

Centre for Diabetes, Endocrinology, and Research, Royal Surrey County Hospital, Guildford, United Kingdom.

**Patients:**

Eight obese men with type 2 diabetes [age, 57.3 ± 1.9 years; body mass index, 30.3 ± 1.0 kg/m^2^; glycosylated hemoglobin, 66.5 ± 2.6 mmol/mol (8.2% ± 0.3%)].

**Interventions:**

Two metabolic studies, 4 weeks after lixisenatide or placebo, with cross-over and repetition of studies.

**Main Outcome Measures:**

Study one: very-low-density lipoprotein (VLDL) and chylomicron (CM) triacylglycerol (TAG) kinetics were measured with an IV bolus of [^2^H_5_]glycerol in a 12-hour study, with hourly feeding. Oral [^13^C]triolein, in a single meal, labeled enterally derived TAG. Study two: glucose kinetics were measured with [U-^13^C]glucose in a mixed-meal (plus acetaminophen to measure gastric emptying) and variable IV [6,6-^2^H_2_]glucose infusion.

**Results:**

Study one: CM-TAG (but not VLDL-TAG) pool-size was lower with lixisenatide (*P* = 0.046). Lixisenatide reduced CM [^13^C]oleate area under the curve (AUC)_60–480min_ concentration (*P* = 0.048) and increased CM-TAG clearance, with no effect on CM-TAG production rate. Study two: postprandial glucose and insulin AUC_0–240min_ were reduced with lixisenatide (*P* = 0.0051; *P* < 0.05). Total glucose production (*P* = 0.015), rate of glucose appearance from the meal (*P* = 0.0098), and acetaminophen AUC_0–360min_ (*P* = 0.006) were lower with lixisenatide than with placebo.

**Conclusions:**

Lixisenatide reduced [^13^C]oleate concentrations, derived from a single meal in CM-TAG and glucose rate of appearance from the meal through delayed gastric emptying. However, day-long CM production, measured with repeated meal feeding, was not reduced by lixisenatide and decreased CM-TAG concentration resulted from increased CM-TAG clearance.

## Teaser

Using stable isotopes, lixisenatide acutely slowed gastric emptying, lowering postprandial TAG levels. A more prolonged effect of reduced chylomicron TAG was from increased clearance.

A substantial postprandial increase in plasma triacylglycerol (TAG) is an independent risk factor for cardiovascular disease (1–4). This can occur via the generation of small, dense, low-density lipoproteins; oxidative stress; inflammation; and/or endothelial dysfunction. An excess of postprandial TAG can result from the overproduction of very-low-density lipoproteins (VLDLs) synthesized by the liver, which contain the higher molecular weight form of apolipoprotein (apo)B, apoB100; chylomicrons (CMs) synthesized in the enterocytes, in response to dietary fat, which contain the lower molecular weight form of apoB, apoB48; impaired TRL clearance; or a combination of these processes.

GLP-1 receptor agonists (RAs) lower postprandial TAG (5–7), which can contribute to their cardiovascular benefit; however, the mechanism has not been elucidated. Insulin resistance is associated with a postprandial increase in CM apoB48 and TAG (8), and some studies have suggested that this results from impaired clearance (9, 10). The small intestine can use endogenous substrates to synthesize TRL particles. For instance, an acute elevation of nonesterified fatty acids (NEFAs) in humans increased basal intestinal apoB48 production (11), and the CM-TAG production rate was shown to be increased in metabolic syndrome (12). Both animal (13) and human (14) studies have suggested that GLP-1 RAs can reduce postprandial TRL-apoB48 independently of their effects on plasma insulin and gastric emptying. In a study of individuals without diabetes, exenatide suppressed the production of apoB48 but with no effect on catabolism (15). However, measurement of apoB48 metabolism provides a measure of particle kinetics rather than the kinetics of the TAG substrate within the particle. No studies have previously investigated the effect of GLP-1 RAs on postprandial CM and VLDL-TAG kinetics. Understanding the regulation of enterocyte lipid handling might provide novel strategies to reduce cardiovascular disease risk.

GLP-1 RAs also effectively lower postprandial hyperglycemia (16–18). This might be achieved through delayed gastric emptying (19, 20), reduction of hepatic glucose output, and increased peripheral glucose uptake (21). Identifying the relative contribution of delayed gastric emptying to glucose regulation will inform therapeutic decision making for those in insulinopenic states and those with pre-existing delayed emptying.

We hypothesized that lixisenatide, a GLP-1 RA, reduces postprandial TAG through a decrease in CM-TAG production, as a result of a direct effect on enterocyte CM assembly, and also from a decrease in VLDL-TAG secretion, owing to an improvement in insulin sensitivity. At the study inception, we hypothesized that GLP-1 RA lowered postprandial glycemia primarily by decreasing endogenous glucose output, although more recent data have suggested that the predominant effect of lixisenatide is via gastric emptying (19).

We used a validated constant-feeding method and three antibody immunoaffinity method (22), which enables complete separation of hepatic TRL from intestinal TRL, to measure hepatic VLDL and intestinal CM-TAG kinetics, with an IV bolus of [^2^H_5_]glycerol. In addition, we used the validated dual-tracer dilution technique to determine glucose appearance after a meal (23).

## Materials and Methods

### Participants

The present study was registered with ClinicalTrials.gov (NCT02049034) and approved by the UK Medicines and Health Care Products Regulatory Agency (EudraCT 2013-002826-22), South Central-Hampshire B National Research Ethics Services Committee, and University of Surrey Ethics Committee. Before the initiation of study procedures, all the participants provided written informed consent. The inclusion criteria were white men with type 2 diabetes, age 40 to 65 years, and receipt of metformin monotherapy (glycosylated hemoglobin, ≥58 but ≤80 mmol/mol; 7.5% to 9.5%). The exclusion criteria were insulin use, oral hypoglycemic agent use (other than metformin), alcohol consumption (>12 g/d of alcohol), current smoking, liver or renal disease, and previous pancreatitis or gastric surgery.

### Experimental design

We performed a double-blind crossover study comparing 4 weeks of an investigational medicinal product (IMP: lixisenatide or placebo) with a 4-week washout period in between (Supplemental Fig. 1). Randomization was performed using SAS, version 9.1, PROC PLAN software (SAS Institute, Cary, NC) prepared by an investigator with no clinical involvement in the trial. The allocation sequence was concealed from the investigators involved in enrolling and assessing the participants using numbered, opaque, sealed envelopes. Lixisenatide was supplied as disposable prefilled pen-injectors for subcutaneous injection: 10-µg lixisenatide in green pens for 14 days and then 20-µg lixisenatide purple pens for an additional 14 days. Placebo for lixisenatide was supplied as corresponding green and purple disposable pen-injectors containing 3 mL of sterile aqueous solution. The injections were self-administered daily, 30 minutes before breakfast, except on the metabolic study days. The participants were required to bring the medication packs to all study visits to verify adherence.

The metabolic studies were performed on 2 days, using two distinct studies (1 and 2), each occurring after completion of 4 weeks of treatment. At all the visits, the subjects attended the Centre for Diabetes, Endocrinology and Research, Royal Surrey County Hospital, Guildford, after an over overnight fast and having consumed a standardized low-fat, low-fiber meal of ~700 kcal at 8 pm the previous evening.

### Study one—postprandial lipid kinetics

The subcutaneous IMP was administered at −270 minutes, 30 minutes before the first of 12 liquid meals at 1-hour intervals (23.8% carbohydrate, 12.8% protein, 63.5% fat; 178 kcal/80 mL) at time −240 minutes. A cannula was inserted into an antecubital vein for blood sampling. Fasting blood samples were taken to measure the fasting glucose, insulin, and lipid profile. The feeding study was designed to increase the fasting plasma TAG levels twofold and to maintain this throughout the study (12). The first 4 hours (−240 to 0 minutes) allowed for achievement of a TAG steady-state before administration of stable isotope tracers. The meal composition (in 80 mL) was 6.5 g of sugar, 7 g of whey powder (Nature’s Best, Tunbridge Wells, United Kingdom), 7 mL of extra virgin olive oil (Tesco, Welwyn Garden City, United Kingdom), 7 mL of sunflower oil (Tesco), 10 mL of double cream (Tesco) and flavoring, prepared as an emulsion.

Each meal, prepared immediately beforehand, was consumed within 1 minute. For the third meal, [1,1,1 ^13^C_3_]triolein (150 mg) was mixed in with the third meal and was consumed at −120 minutes to investigate the acute effect of the IMP on TAG absorption from the meal. An IV bolus of [^2^H_5_]glycerol (0.75 µmol/kg) was administered at 0 minutes. Blood samples were taken at regular intervals until 480 minutes for measurement of the isotopic enrichment of CM and VLDL-TAG with [^2^H_5_]glycerol and [^13^C]oleate (Supplemental Fig. 2).

### Study two—postprandial glucose kinetics

One indwelling cannula was inserted into an antecubital vein of each arm. Fasting blood was sampled for insulin, glucose, cholesterol, TAG, and NEFA concentrations. A primed, IV infusion of [6,6-^2^H_2_]glucose (6 mg/kg; 0.06 mg/kg/min) was administered from −120 to 0 minutes, followed by a variable infusion to 360 minutes, to measure endogenous glucose production (EGP) (23).

The subcutaneous IMP was administered at −30 minutes. At 0 minutes, a liquid mixed meal (64% carbohydrate, 14.4% protein, 21.6% fat; 500 kcal in total) containing [U-^13^C] glucose (1.7 g) was given to measure the meal-derived glucose appearance. The [6, 6-^2^H_2_] glucose infusion rate was adjusted at predetermined intervals to mimic the (expected) EGP (Supplemental Fig. 3). Blood samples were taken at regular intervals for 360 minutes to measure glucose enrichment and the concentrations of glucose, TAG, NEFA, and insulin. The participants were asked to void before the meal. During the study and at study termination, the urinary volume and glucose concentration were measured, as necessary. At 0 minutes, 1000 mg acetaminophen dissolved in 30 mL of water was given immediately before the meal to measure gastric emptying.

#### Determination of glucose enrichment in plasma

The samples were deproteinized and dried under oxygen-free nitrogen. Glucose in the sample was derivatized to form a methoxime-trimethylsilane derivative, and enrichment was measured using gas chromatography mass spectrometry (GCMS; Agilent 5975C; Agilent Technologies, Santa Clara, CA) in the electron ionization (EI) mode. The ions monitored were at *m/z* 319.2, *m/z* 321.2 (m+2), *m/z* 322.2 (m+3), and m*/z* 323.2 (m+4) (24). The intra-assay CVs for determination of isotopic enrichment of glucose in *m/z* 321.2/319.2 at three enrichment levels (low, middle and high) were 0.33%, 0.33%, and 0.25% and for *m/z* 323.2/319.2 were 1.99%, 0.3%, and 0.2%, respectively.

#### Lipoprotein separation and isolation

TRL particles [Svedberg flotation (SF) >20] were isolated from plasma overlaid by saline containing 0.1% EDTA (w/v) by floatation ultracentrifugation in a fixed angle rotor (50.4Ti; Beckman, High Wycombe, United Kingdom) using an LE80-k ultracentrifuge (Beckman Coulter Optima, High Wycombe, United Kingdom) at 125,000*g* for 16 hours at 4°C.

VLDL and CM particles were isolated from the TRL samples using a sequential immunoaffinity binding method, as previously described (12, 22). Three monoclonal antibodies to apoB100, 4G3, 5E11, and 16BSol (Heart Institute, University of Ottawa, Ottawa, ON, Canada) coupled separately to protein G Sepharose 4 Fast flow (Amersham, United Kingdom) were used sequentially. The bound apoB100-containing VLDL fractions from the sequential affinity chromatography were combined, and the unbound apoB48-containing CM fractions were also combined.

### Determination of plasma-free glycerol and TAG-glycerol enrichment

For analysis of plasma-free glycerol, the samples were deproteinized and further purified using ion-exchange chromatography. Freeze-dried glycerol was derivatized with MTBSTFA to form TBDMS glycerol, and enrichment was measured using GCMS in the EI mode. The ions monitored were *m/z* 377.4 and *m/z* 382.4 (m+5) (25). TAG in VLDL and CM fractions was extracted and hydrolyzed in the presence of 3% hydrochloric acid/methanol to glycerol and methylesters of fatty acids. Glycerol from TAG was then purified by ion exchange and freeze dried, and enrichment was measured using GCMS (Agilent 5973 network MSD; Bracknell, United Kingdom) in EI mode after derivatization to TBDMS glycerol. The intra-assay CVs for plasma glycerol low, middle, and high were 8.8%, 8.0%, and 5.1%, respectively. The intra-assay CVs for TAG glycerol low, middle, and high were 12.6%, 3.7%, and 4.6%, respectively.

#### Determination of oleate enrichment of VLDL and CM-TAG and TAG concentration

Enrichment of VLDL and CM-TAG with [^13^C]oleate was measured using Trace GC Ultra with an autosampler, coupled to an isotope ratio mass spectrometer (Delta Plus XP; Thermo Electron Corporation, Bremen, Germany) via an oxidation reactor, reactor temperature 960°C, and combustion interface III.

An internal standard, heptadecanoic acid, added to the TAG samples at the acid hydrolysis and esterification step, was used to measure the concentration of VLDL-TAG and CM-TAG. The ApoB48 concentration was measured using a commercially available kit (Shibayagi, Co., Ltd., Shibukawa, Japan).

#### Measurement of lipid and hormone concentrations

The concentrations of fasting and fed plasma NEFA, TAG, total cholesterol, high-density lipoprotein cholesterol, and TRL-TAG and TRL cholesterol were measured using an enzymatic assay (ABX; Chicksands, Shefford, United Kingdom) using Cobas MIRA (Roche, Welwyn Garden City, United Kingdom). The insulin concentrations were measured using radioimmunoassay (Millipore Corporation, Billerica, MA).

#### Data analysis

All tracer enrichments are expressed as the tracer/tracee ratio (TTR). Glucose fluxes were calculated from the TTR and native glucose using the Mari model (26) implemented within the Bayesian parameter estimation framework (23).

Using compartmental modeling (Supplemental Fig. 4), chylomicron-TAG and VLDL-TAG clearance and production rates were analyzed (27). A single-pool model was used to describe CM-TAG and VLDL-TAG kinetics with plasma glycerol as precursor pool using the SAAM ΙΙ program (SAAM Institute, Seattle, WA). The model represents the kinetics of the TTR profiles, which change as labeled glycerol is removed from plasma and incorporated into the TAG fractions. Plasma glycerol kinetics were described by a sum of three exponentials representing a three-compartment model. A five-compartment chain described a time delay due to synthesis and secretion of VLDL-TAG or CM-TAG.

The model assumes steady-state of native (unlabeled) glycerol throughout the experimental period (*i.e.,* a constant appearance, disappearance, and incorporation of native glycerol into the TAG fractions). The incorporation of glycerol into VLDL by the liver and the intestine is subject to a delay. The VLDL-TAG and CM-TAG production rates were calculated as the product of the VLDL-TAG fractional clearance rate (FCR) and CM-TAG FCR and their respective TAG pools. VLDL and CM-TAG pools were calculated from the VLDL and CM-TAG concentration and plasma volume, which was determined using the method of Pearson *et al.* (28).

The CM [^13^C]oleate concentration was calculated by multiplying the CM oleate TTR by the CM-TAG concentration. The homeostasis model assessment (HOMA) index 2 of insulin resistance (HOMA2-IR) was calculated using the HOMA calculator, version 2.2 (29). The Matsuda index of insulin sensitivity was calculated using insulin values (µU/mL) and glucose values (mg/dL) obtained from the single mixed-meal in study one: Matsuda index = 10,000/√ (fasting glucose × fasting insulin) (mean glucose × mean insulin) (30–32).

### Statistical analysis

A general linear mixed model with repeated measures was used with SAS PROC MIXED and explanatory variables of period and treatment. The variance covariance matrix used for the repeated measure time course measurements was SP(POW). Participant was a random effect. For measurements, such as weight, which were measured twice for each period, the difference at each period was analyzed in a two-period cross-over model, with period and treatment as independent variables and participant as a random effect. An analysis was conducted with the period-by-treatment interaction included as an explanatory variable. Significance was accepted at the 5% level, without the use of multiplicity adjustment. Results are presented as mean ± SEM.

### Determination of sample size

The sample size was based on the primary endpoint of the total rate of glucose appearance after the breakfast meal. This was calculated using data from a study measuring the effect of exenatide on postprandial total glucose rate of appearance (Ra) in the presence of type 2 diabetes (16). In the present study, the total glucose Ra area under the curve (AUC) was 23.7 ± 3.0 µmol/kg/min (mean ± SD) in participants before treatment and 14.3 ± 3.5 µmol/kg/min after 2 weeks of treatment. The correlation between measurements in the same person was unknown and, thus, was assumed to be zero (worst case scenario). Completing the study in 12 participants would have provided 80% power to detect a difference of 20%.

The power calculation was recalculated using the data from the eight participants who finished the trial, based on the primary endpoint of total glucose Ra AUC after the meal (as described). Completing the study in eight participants provided 80% power to detect a difference of 26%. The correlation between measurements in the same person was unknown and, thus, was assumed to be zero (worst case scenario). This was, therefore, a conservative estimate. In 2011, Cersosimo *et al.* (16) found a 40% difference in the glucose Ra AUC after 2 weeks of treatment.

## Results

### Demographic data and biochemistry measurements

Eight men, aged 57.3 ± 1.9 years, were studied. Recruitment was from May 2014 to January 2016. The participant flow diagram is shown in Supplemental Fig. 5. No serious adverse events occurred. Six of the participants were taking HMG coenzyme A reductase inhibitors. No differences were found between, or within, the lixisenatide and placebo treatment phases at baseline or at the end of treatment, for all demographic and fasting biochemistry measurements (Table 1). After 4 weeks of IMP, the HOMA2-IR was not significantly different between the lixisenatide and placebo groups. The Matsuda index was higher with lixisenatide (4.4 ± 2.0 vs 3.5 ± 2.5; *P* = 0.011).

**Table 1.** Participant Characteristics at Baseline and End of Treatment With Either Lixisenatide or Placebo

Characteristic	Lixisenatide		Placebo	
	Baseline	End	Baseline	End
BMI, kg/m^2^	30.0 ± 1.2	29.2 ± 1.3	30.1 ± 0.9	29.6 ± 1.1
Body weight, kg	91.8 ± 3.4	89.4 ± 3.3	92.2 ± 2.9	90.7 ± 3.1
Fat-free mass, kg	66.3 ± 2.1	65.3 ± 1.9	66.0 ± 2.1	65.9 ± 2.0
Amylase, U/L	46.4 ± 5.9	45.8 ± 5.4	45.4 ± 4.9	42.3 ± 5.2
ALT, IU/L	44.5 ± 7.9	41.8 ± 6.8	41.6 ± 6.3	43.9 ± 9.0
Total plasma cholesterol, mmol/L)	3.9 ± 0.3	3.5 ± 0.3	4.1 ± 0.4	4.0 ± 0.3
Fasting plasma TAG, mmol/L	1.68 ± 0.3	1.69 ± 0.2	1.72 ± 0.2	1.59 ± 0.2
HbA1c, mmol/mol (%)	61.5 ± 3.2 (7.8 ± 0.3)	55.1 ± 3.0 (7.2 ± 0.3)	63.4 ± 3.5 (8.0 ± 0.3)	62.6 ± 4.0 (7.9 ± 0.3)
Lipase, U/L	34.5 ± 2.6	40.6 ± 4.3	36.8 ± 4.3	37.1 ± 3.2
Calcitonin, ng/L	4.3 ± 0.4	2.9 ± 0.7	3.12 ± 0.5	2.4 ± 0.5

No statistically significant differences were found within or between treatments in these measures.

Abbreviations: ALT, alanine aminotransferase; BMI, body mass index; HbA1c, glycosylated hemoglobin.

### Lipid metabolic study (study one)

**Figure 1.**
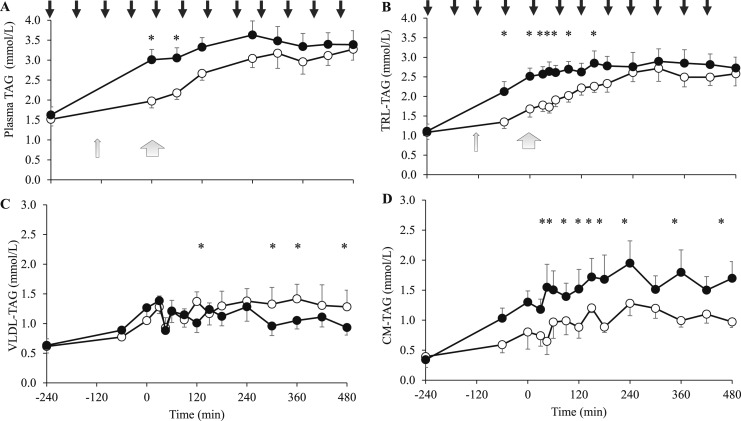
(A) Plasma TAG, (B) TRL-TAG, (C) VLDL-TAG, and (D) CM-TAG concentrations (mmol/L) at fasting (−240 minutes) and after meal drinks at 1-hour intervals (−240 to 480 minutes) at the end of 4 weeks of treatment with either lixisenatide (white circles) or placebo (black circles) (study one). Lixisenatide or placebo injection was self-administered at −270 minutes. The results are presented as mean ± SEM. Black arrows indicate hourly meals; narrow gray arrows, the meal labeled with [1,1,1 ^13^C_3_]triolein; and wide gray arrows, the bolus of [1,1,2,3,3 ^2^H_5_]glycerol administered at the start of steady-state. *P < 0.05.

No differences were found in the fasting plasma TAG or TRL-TAG concentrations at −240 minutes or in the mean steady-state concentrations between treatments (Fig. 1A and 1B), although both were slower to increase from fasting values with lixisenatide than with placebo. A steady-state in the apoB48 concentration was achieved in the lixisenatide (mean, 8.70 mg/L; 95% CI, 7.11 to 10.29 mg/L; *P* = 0.510) and placebo (mean, 12.12 mg/L; 95% CI, 9.59 to 14.65 mg/L; *P* = 0.957) studies both. The VLDL-TAG pool size and mean postprandial VLDL-TAG concentration were not different between treatments, although the postprandial VLDL-TAG was greater at four time points with lixisenatide (Fig. 1C; Supplemental Table 1). The mean postprandial CM-TAG concentration and pool size were lower with lixisenatide compared with placebo (*P* = 0.043 and *P* = 0.047, respectively; Fig. 1D).

**Figure 2.**
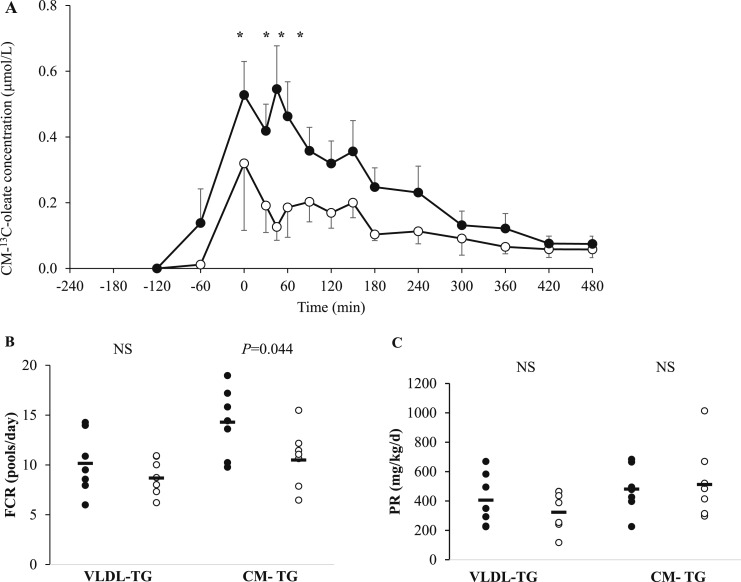
(A) CM [^13^C]oleate concentration (µmol/L) at fasting (−240 minutes) and after meal drinks at 1-hour intervals (−240 to 480 minutes) at the end of 4 weeks of treatment with either lixisenatide (white circles) or placebo (black circles) (study one). [^13^C]oleate was mixed with the third meal (−120 minutes). The lixisenatide or placebo injection was self-administered at −270 minutes. (B) FCR of VLDL-TAG and CM-TAG (pools per day). (C) Production rate of VLDL-TAG and CM-TAG (mg/d/kg body weight). The results are presented as mean ± SEM. *P < 0.05.

The CM-[1-^13^C]oleate concentration AUC_60–480min_ (Fig. 2A) was reduced after lixisenatide compared with after placebo (*P* = 0.048). The CM-TAG FCR, a measure of clearance, was significantly greater with lixisenatide than with placebo (*P* = 0.044; Fig. 2B). However, the CM-TAG production rate was not different between treatments (Fig. 2C), and the VLDL-TAG FCR and production rate were not different between treatments (Fig. 2B and 2C).

### Glucose metabolic study (study two)

**Figure 3.**
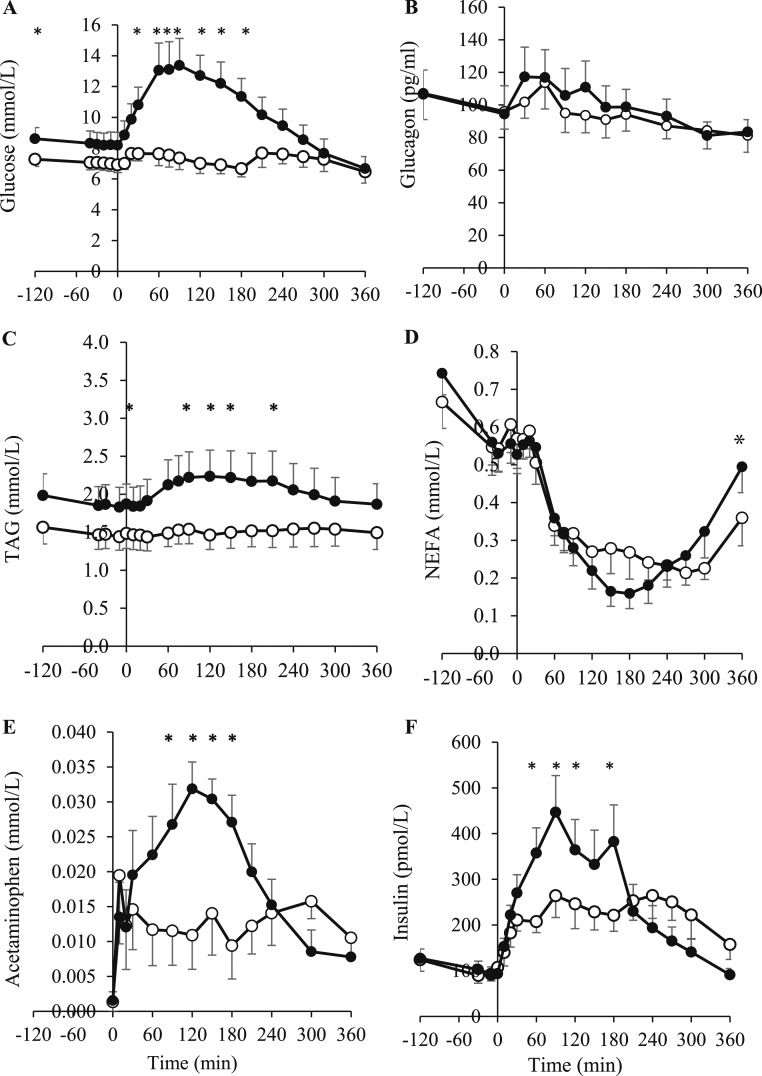
(A) Plasma glucose, (B) glucagon, (C) TAG, (D) NEFA, (E) acetaminophen, and (F) insulin (pmol/L) at fasting (−120 to 0 minutes) and in response to a mixed meal and acetaminophen (consumed at 0 minutes) at the end of 4 weeks of treatment with either lixisenatide (white circles) or placebo (black circles) (study two). The lixisenatide or placebo injection was self-administered at −30 minutes. The results are presented as mean ± SEM. *P < 0.05.

The glucose concentration in response to a mixed-meal consumed at 0 minutes is shown in Fig. 3A. The fasting glucose concentration at time 0 minutes and AUC_0–240min_ were significantly lower after lixisenatide (*P* = 0.020 and *P* = 0.004, respectively). The glucagon concentration was not different between treatments (Fig. 3B). Plasma TAG AUC_0–180min_, corrected for fasting values, was significantly lower with lixisenatide than with placebo (*P* = 0.021; Fig. 3C). However, the NEFA AUC was not significantly different (Fig. 3D). The acetaminophen AUC_0–360min_ was lower with lixisenatide than with placebo (*P* = 0.006; Fig. 3E). The insulin concentration AUC_0–180min_ and AUC_0–240min_, were significantly lower with lixisenatide than with placebo (*P* = 0.024 and *P* = 0.045, respectively; Fig. 3F) although the AUC_0–360min_ was not different.

**Figure 4.**
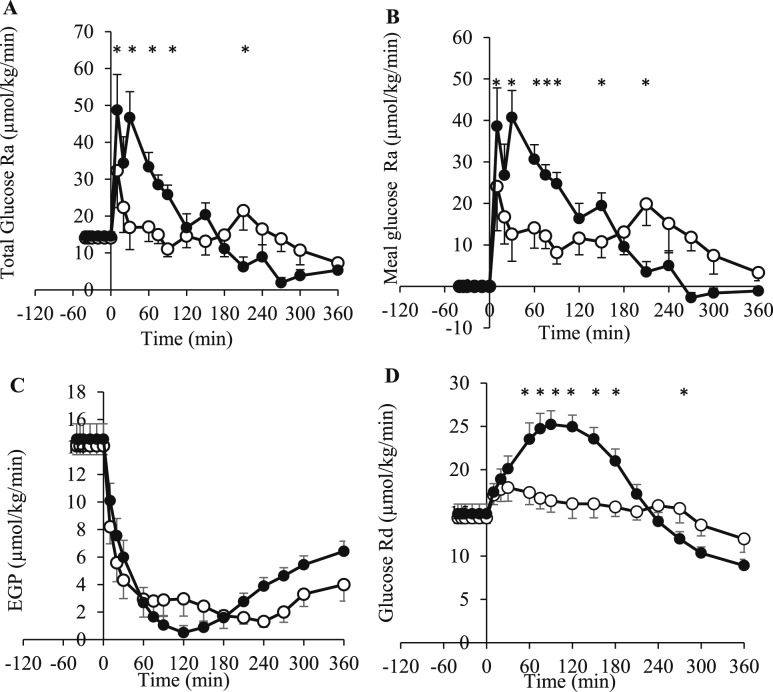
(A) Total glucose Ra, (B) meal glucose Ra, (C) EGP, and (D) Rd (µmol/kg/min) at fasting (−40 to 0 minutes) and in response to a mixed meal (consumed at 0 minutes) at the end of 4 weeks of treatment with either lixisenatide (white circles) or placebo (black circles) (study two). The lixisenatide or placebo injection was self-administered at −30 minutes. The results are presented as mean ± SEM. *P < 0.05.

After the meal, the total glucose Ra AUC_0–240min_ was lower with lixisenatide (*P* = 0.002; Fig. 4A). The glucose Ra from the meal, AUC_0–240min_, was lower with lixisenatide than with placebo (*P* = 0.013; Fig. 4B).

The fasting EGP was not different between treatments. The EGP decreased from fasting values after consumption of the mixed meal, with no difference between treatments (Fig. 4C). However, the EGP AUC_0–180min_ and AUC_0–240min_, corrected for fasting values, were lower with placebo (*P* = 0.032 and *P* = 0.049). The EGP AUC_0–360min_ was not different with or without correction for fasting values.

The fasting glucose disposal rate (Rd) was not different between treatments. The glucose Rd was greater after placebo between 60 and 180 minutes and lower at 270 minutes (*P* = 0.039; Fig. 4D). The glucose Rd AUC_0–240min_ was lower with lixisenatide (*P* = 0.005). The fasting glucose metabolic clearance rate (MCR) was greater with lixisenatide than with placebo (*P* = 0.013). The glucose MCR AUC_0–360min_ was also greater with lixisenatide (*P* = 0.008), although the difference was not statistically significant once corrected for the fasting values (data not shown).

## Discussion

The major finding of the present study was of a prolonged effect of lixisenatide (assessed using a 12-hour repeated feeding protocol) to reduce the CM-TAG concentration through increased clearance. However, acutely, after a single meal, the dietary TAG appearance was reduced, as shown by the delayed appearance of [^13^C]oleate in CM-TAG.

Our experimental protocol was designed to examine the postprandial TAG metabolism by (i) the appearance of [^13^C]oleate in plasma TAG after a high-fat 80-kcal single meal labeled with [^13^C]triolein, given 150 minutes after injection of the IMP (study one); (ii) hourly feeding of high-fat 80-kcal single meals for 12 hours to achieve a steady-state in plasma TAG (study one) and measurement of CM- and VLDL-TAG kinetics; and (iii) the plasma TAG response to a standard 500-kcal liquid meal in study two.

The addition of [^13^C]triolein to the third meal, in study one, demonstrated reduced fatty acid absorption with lixisenatide with fat-rich feeding. Delayed gastric emptying has previously been shown with lixisenatide after a high-fat meal (19). Similarly, it was evident from study two that lixisenatide led to a near abolition of the postprandial TAG increment—a similar effect to that reported previously with exenatide (5, 7).

The hourly meal-feeding design addressed whether lixisenatide has a prolonged effect on the VLDL and CM metabolism, eliminating any appetite-dependent effects of GLP-1 on plasma TAGs. With a Western diet, people will have elevated TAGs throughout the day; thus, repeated meal feeding might approximate a usual physiological state (33).

To the best of our knowledge, our study is the first report that a GLP-1 RA improves postprandial TAG and TRL-TAG concentrations by reducing the circulating CM-TAG concentration via an increase in the CM-TAG FCR. No previous study has measured the effect on CM-TAG kinetics. The uniqueness of the present study was the ability to separate CM-TAG from VLDL-TAG using immunoaffinity. It has been reported that GLP-1 reduces postprandial CM particle production (15, 34) but with no effect on CM particle clearance (15). In a study of healthy adults by Xiao *et al.* (15), a steady-state in TRL-apoB48 concentrations was not achieved, which meant that the steady-state modeling used carried a degree of imprecision. We found no change in the apoB48 concentration during the steady-state in the present study. Our participants had type 2 diabetes, which could also have accounted for the differences in outcomes. The statistical significance in our data with eight subjects suggests a large effect size, and the crossover design used ensured the tightest control on variability such that the comparisons between treatments were performed within subjects to maximize the available power. We studied white men, which could have limited the generalizability of the data to other groups.

In the repeated feeding study, the IMP was administered during consumption of the first meal at −270 minutes. The drug concentrations of lixisenatide peak after 1 to 2 hours, and it has a half-life of 2 to 4 hours (35, 36). Although the high affinity of lixisenatide for the GLP-1 receptor might allow for a more persistent metabolic response (37), a waning drug effect could explain the discrepancy between the lack of effect of lixisenatide on the CM production rate (measured during the repeated feeding study) and the reduction in CM [^13^C]oleate after a single meal at −120 minutes.

The effect of lixisenatide to increase CM clearance might have resulted from an increase in lipoprotein lipase (LPL) activity. The control of LPL expression and action is complex and is tissue specific; however, includes regulation by insulin and glucagon (38), both of which can be affected by GLP-1 (39, 40). We did not find statistically significant differences in these hormones. LPL is also negatively regulated by apoCIII, and it has been shown that a single injection of exenatide can reduce the postprandial elevation of apoCIII (7). Microvascular recruitment could also facilitate the clearance of CM-TAG (40, 41), and this mechanism requires further study.

We hypothesized that lixisenatide would decrease VLDL-TAG production owing to improved insulin sensitivity. After 4 weeks of lixisenatide, the Matsuda Index (a measure of whole body insulin sensitivity) had improved significantly. However, HOMA2-IR (considered to represent hepatic sensitivity) did not change, which might explain the lack of effect on hepatic VLDL-TAG production (42).

At study inception, it was postulated that lixisenatide resulted in a multimodal reduction of postprandial glucose (43). However, we found that the predominant mechanism by which lixisenatide reduces postprandial glycemia was through a reduction of glucose Ra from the meal—itself a consequence of delayed gastric emptying. We found that lixisenatide had no additional suppressive effect on EGP from that observed with placebo. Our data, therefore, have confirmed that slowing gastric emptying is the primary mechanism for prandial glucose regulation with lixisenatide (19, 44).

The experimental protocol was not designed to match the insulin and glucagon concentrations between the study arms. Just as with other GLP-1 RAs, lixisenatide stimulates glucose-dependent insulin secretion by pancreatic *β*-cells (39). In our study, lixisenatide ameliorated postprandial hyperglycemia despite a lower increment in insulin concentrations than with placebo. Delayed gastric emptying would result in diminished nutrient appearance in the circulation, thereby reducing *β*-cell stimulation (39). Even so, the insulin response (relative to the glycemic stimulus) after lixisenatide was considerable.

Glucagon secretion can be suppressed by GLP-1 RAs (including lixisenatide) (39); however, our data suggest that, in the context of delayed gastric emptying, inhibition of glucagon secretion contributes little to suppression of endogenous glucose Ra with lixisenatide. This is commensurate with data from a pancreatic clamp protocol, in which GLP-1 infusion inhibited EGP independently of insulin and glucagon (45).

The acetaminophen profiles indicated marked slowing of gastric emptying with lixisenatide for ≤3 hours after the meal. Short-acting GLP-1 RAs, such as lixisenatide, have a more pronounced inhibitory effect on gastric emptying than longer acting GLP-1 RAs, with the latter also showing tachyphylaxis with prolonged use (19, 46). Once-daily (morning) lixisenatide achieved better postprandial glucose control at breakfast compared with liraglutide (21) but with progressively less differentiation of postprandial glucose control throughout the day (47). Hypothetically, progressive lessening of the inhibitory action of lixisenatide on gastric emptying could be responsible for this pattern.

The increase in fasting glucose MCR with lixisenatide potentially relates to improved peripheral insulin sensitivity. Whether GLP-1 RAs have direct action on skeletal muscle to improve glucose clearance is uncertain. This has been suggested to occur with exenatide and liraglutide in studies using myotubes (48); however, no effect was seen in a murine model (49) or when using a pancreatic clamp with GLP-1 infusion in patients with type 2 diabetes (50). In healthy subjects, GLP-1 induces vasodilation in adipose tissue and skeletal muscle (40); thus, it is possible that microvascular recruitment facilitates clearance.

In conclusion, we have shown that the short-acting GLP-1 RA, lixisenatide, ameliorates postprandial TAG and glucose after a single meal through delayed gastric emptying. However, a prolonged effect of lixisenatide to reduce CM-TAG was also observed, which was due to increased clearance, demonstrating that lixisenatide mediates the metabolic effects independently of an effect on gastric emptying.

## Supplementary Material

Supplemental Data

## Abbreviations:

apo: apolipoprotein
AUC: area under the curve
CM: chylomicron
CV: coefficient of variation
EGP: endogenous glucose production
EI: electron ionization
FCR: fractional clearance rate
GCMS: gas chromatography mass spectrometry
GLP-1: glucagon-like peptide-1
HOMA: homeostasis model assessment
HOMA2-IR: HOMA index 2 of insulin resistance
IMP: investigational medicinal product
LPL: lipoprotein lipase
MCR: metabolic clearance rate
NEFA: nonesterified fatty acid
Ra: rate of appearance
RA: receptor agonist
Rd: disposal rate
TAG: triacylglycerol
TRL: triglyceride-rich lipoprotein
TTR: tracer/tracee ratio
VLDL: very low density lipoprotein
