# Supplementary material for: Lixisenatide Reduces Chylomicron Triacylglycerol by Increased Clearance
Source: J Clin Endocrinol Metab. 2018 Sep 11;104(2):359–68. doi: 10.1210/jc.2018-01176 (PMC6300412; doi:10.1210/jc.2018-01176)
Supplement: Supplemental Data [file jc.2018-01176.sd1.docx]

**0**  28 56 84 days

**Period 1 Period 2**

**4 weeks**

**washout**

Placebo

Lixisenatide

Lixisenatide

Placebo

4 weeks

4 weeks

**Supplementary Figure 1.** Schematic of the trial design

**Bolus: [1,1,2,3,3,^2^ H_5_]Glycerol 0.75µmol/kg)**

Spiked Meal

[1,1,1 ^13^C_3_]triolein

-240 -120 0 120 240 360 480 min

**Supplementary Figure 2.** Schematic of Study one

Down-pointing light grey arrows indicate hourly meals. Up-pointing black arrows indicate blood sampling points


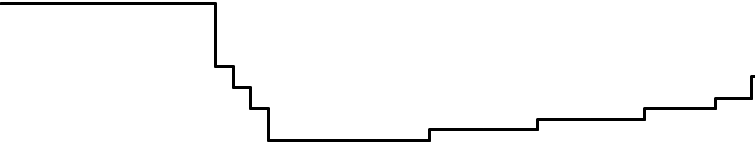


-120 -30 0 120 240 360min

Mixed meal spiked with [U-^13^C] glucose

IMP

Primed (6mg/kg); variable infusion [6,6 ^2^H_2_] glucose

Parac

**Supplementary Figure 3**. Schematic of Study two.

IMP: Investigational medicinal product administered subcutaneously; Para: paracetamol (acetaminophen) taken orally. Up-pointing black arrows indicate blood sampling points


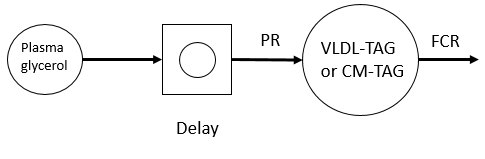


**Supplementary Figure 4**. Multi-compartmental model for CM and VLDL TAG kinetics.

CM: chylomicron; FCR: fractional clearance rate; PR: production rate; TAG: triacylglycerol; VLDL: very low-density lipoprotein

**Supplementary Table 1.** Postprandial VLDL & CM kinetics following hourly meal drinks (from -240min to 480 min; study one).

|  | **Lixisenatide** | **Placebo** | ***P* value** |
| --- | --- | --- | --- |
| CM-TAG pool-size baseline (mg) | 1151.3 ± 519.3 | 1004.6 ± 246.4 | 0.731 |
| CM-TAG pool-size postprandial (mg) | 2682.9 ± 457.1 | 4589.5 ± 707.6 | 0.047 |
| CM-TAG PR (mg/day/kg BW) | 481 ± 60 | 512 ± 83 | 0.238 |
| CM-TAG FCR (pools/day) | 14.3 ± 1.3 | 10.5 ± 1.0 | 0.046 |
| VLDL-TAG pool-size baseline (mg) | 1834.2 ± 414.6 | 1870.8 ± 377.8 | 0.950 |
| VLDL-TAG pool-size postprandial (mg) | 3797.4 ± 3385.9 | 3385.9 ± 492.2 | 0.272 |
| VLDL-TAG PR (mg/day/kg BW) | 406 ± 67 | 323 ± 47 | 0.137 |
| VLDL-TAG FCR (pools/day) | 10.2 ± 1.2 | 8.7 ± 0.6 | 0.262 |

BW: body weight, CM-TAG: chylomicron triacylglycerol, FCR: fractional clearance rate, PR: production rate, VLDL-TAG: very low density lipoprotein triacylglycerol
